# Fatigue Crack Growth Analysis under Constant Amplitude Loading Using Finite Element Method

**DOI:** 10.3390/ma15082937

**Published:** 2022-04-18

**Authors:** Abdulnaser M. Alshoaibi

**Affiliations:** Mechanical Engineering Department, Faculty of Engineering, Jazan University, P.O. Box 114, Jazan 45142, Saudi Arabia; alshoaibi@jazanu.edu.sa

**Keywords:** fatigue analysis, equivalent stress intensity factor, linear elastic fracture mechanics, ANSYS, constant amplitude loading

## Abstract

Damage tolerant design relies on accurately predicting the growth rate and path of fatigue cracks under constant and variable amplitude loading. ANSYS Mechanical R19.2 was used to perform a numerical analysis of fatigue crack growth assuming a linear elastic and isotropic material subjected to constant amplitude loading. A novel feature termed Separating Morphing and Adaptive Remeshing Technology (SMART) was used in conjunction with the Unstructured Mesh Method (UMM) to accomplish this goal. For the modified compact tension specimen with a varied pre-crack location, the crack propagation path, stress intensity factors, and fatigue life cycles were predicted for various stress ratio values. The influence of stress ratio on fatigue life cycles and equivalent stress intensity factor was investigated for stress ratios ranging from 0 to 0.8. It was found that fatigue life and von Mises stress distribution are substantially influenced by the stress ratio. The von Mises stress decreased as the stress ratio increased, and the number of fatigue life cycles increased rapidly with the increasing stress ratio. Depending on the pre-crack position, the hole is the primary attraction for the propagation of fatigue cracks, and the crack may either curve its direction and grow towards it, or it might bypass the hole and propagate elsewhere. Experimental and numerical crack growth studies reported in the literature have validated the findings of this simulation in terms of crack propagation paths.

## 1. Introduction

One of the most common catastrophic failures in mechanical structures is fatigue. Over the last few decades, researchers have strived to comprehend the mechanism of fatigue loading in materials that were exposed to dynamic loading, starting with the stress and strain life methodologies proposed by many researchers [1,2,3,4,5,6], which were curve-fitting-based approaches that used nominal and local stress–strain values. Another approach is the energy-based approach proposed by [7], which has since been used as the starting point for several experimental studies. However, such techniques are mostly limited to calculating the permissible number of load cycles before material failure instead of presenting characteristics of fatigue crack nucleation and propagation mechanisms. To properly study fatigue failure, several parameters, such as stress level, loading frequency, stress ratio (R = min/max), and material type, must be considered. In several studies, it has been demonstrated that the level of stress applied has a major impact on the fatigue failure of materials [8,9,10,11]. The linear elastic fracture mechanics (LEFM) theory was developed to identify the issue of fatigue crack growth [12,13,14]. The LEFM methods are commonly adopted for use on long cracks within small-scale yielding. The LEFM techniques are commonly used on long fractures with small-scale yielding behaviors near the crack tip, i.e., the Paris regime near the crack tip, i.e., the Paris regime [12,15]. Meanwhile, the Boundary Element Method [16,17], Meshless Method [18], Finite Difference Method [19], Finite Element Method (FEM), and Extended Finite Element Method (XFEM) [20,21,22] are the most used methodologies for modeling crack propagation. The most common computational approach for simulating damage and failure under both static and dynamic loadings is the FEM, which obtained stress, strain, displacement, and stress intensity factor (SIF) solutions for a wide range of engineering problems. The FEM, commonly known as adaptive remeshing procedures, has proved to be highly effective and reliable. The adaptive remeshing procedures consist of four main steps: (1) existence of a demonstrative 3D finite element framework; (2) prediction of the equivalent SIFs along the crack front; (3) prediction of crack front progressions using appropriate fatigue crack growth law; and (4) specification of a new 3D finite element model considering the new crack front. These procedures are continued until a predetermined crack length or ultimate fracture is reached. Using the 3D FEM to compute the stress intensity factor at a set of points on the crack front, the fatigue crack growth analysis can be accomplished precisely. Nowadays, there is a variety of software to deal with the problem of fatigue crack growth, e.g., FRANC3D [23], ABAQUS [24], ANSYS [25,26,27,28,29,30], ZENCRACK [31], COMSOL [32], BEASY [33], and NASTRAN [34]. Three approaches have been commonly used to describe material fatigue analysis: the method of fracture mechanics proposed by Paris and Erdogan [35], the method of strain–life introduced by Coffin [36], and the method of stress–life introduced by Wöhler [37]. In this work, the first technique was used to estimate fatigue life, in which the crack tip was entirely described by the stress intensity factors. Various experimental procedures have been reported; however, the procedures are generally time-consuming and costly to implement. A numerical analysis approach such as the ANSYS Mechanical R19.2 is an effective process to save both time and money in the laboratory by reducing the amount of work, time, and expenses. Alternatively, there was also an analytical-based technique that was efficient in simulating fatigue growth [38,39]. The main motivation for this study was to make a significant contribution to the use of ANSYS as an effective tool for simulating crack growth under mixed-mode loading situations and monitoring the influence of the holes and crack location on the crack growth trajectory.

## 2. SMART Crack Growth Procedure

SMART is an efficient fracture mechanics simulation approach based on an adaptive meshing strategy in the surrounding area of the crack propagation path. At a certain loading level, the crack begins to grow as soon as a critical value is reached. The crack propagates either to a certain limit specified by the user or to the point where generating a new mesh is impossible, which generally corresponds to the total split of the body into sections. The Unstructured Mesh Method (UMM) was employed in ANSYS to reduce the consumption time in the pre-processing using the tetrahedral mesh generated automatically for the crack front instead of using the ideal hex mesh configuration, reducing the computational time from a few days to a few minutes. The UMM approach is described in detail in [40]. Tetrahedron meshes were used for the crack fronts in the SMART analysis, which were automatically updated as the crack front changed due to the crack growth. The crack propagation path is defined by an angle θ, which is estimated by the ratio of modes of SIF at the crack tip [41,42,43]. A mixed-mode loading condition is considered by ANSYS, and the maximum circumferential stress is used as a crack growth criterion in the present study. Based on this criterion, the following formula is used for the crack growth path in ANSYS [25,44]:

(1) $$\theta =\cos^{-1}\frac{3{\left(K_{II}^{\max}\right)}^{2}+(K_{I}^{\max})\sqrt{{\left(K_{I}^{\max}\right)}^{2}+8{\left(K_{II}^{\max}\right)}^{2}}}{{\left(K_{I}^{\max}\right)}^{2}+9{\left(K_{II}^{\max}\right)}^{2}}$$

where:

$K_{I}^{\max}$ = maximum values of the first mode of SIF under cyclic loading, and

$K_{II}^{\max}$= maximum values of the second mode of SIF under cyclic loading.

The SIFs were calculated via interaction-integral evaluation at the solution phase of the analysis, and then the values were stored in the results file. The crack propagation simulation in this ANSYS simulation is confined to region II of the typical crack propagation under fatigue loading, which may be expressed as:

(2) $$\frac{da}{dN}=C{\left(\Delta K_{eq}\right)}^{m}$$

where *a* = crack length, *n* = the number of fatigue life cycles, *C* = Paris constant, *m* = Paris exponent, and $\Delta K_{eq}$= the equivalent range of stress intensity factor, which may be represented as [44,45]:

(3) $$\Delta K_{eq}=\frac{1}{2}\cos\left(\frac{\theta }{2}\right)\left[\Delta K_{I}(1+\cos\theta )-3\Delta K_{II}\sin\theta \right]$$

where:

(4) $$\begin{array}{l} \Delta K_{I}=K_{I}^{\max}-K_{I}^{\min}=(1-R)K_{I}^{\max}\\ \Delta K_{II}=K_{II}^{\max}-K_{II}^{\min}=(1-R)K_{II}^{\max} \end{array}$$

as R represents the load ratio.

According to Equation (2), with a crack growth increment Δ*a*, the fatigue life cycles can be expressed as:

(5) $$\int_{0}^{\Delta a}\frac{da}{C{\left(\Delta K_{eq}\right)}^{m}}=\int_{0}^{\Delta N}dN=\Delta N$$

**Figure 1:**
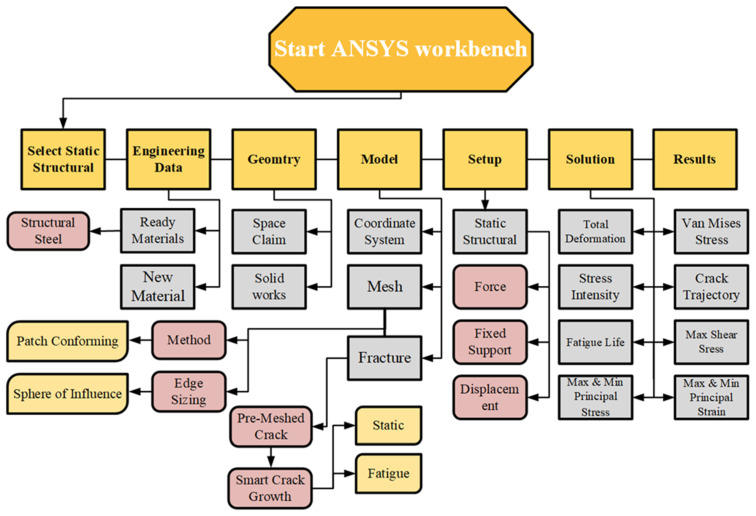
Flowchart for the ANSYS SMART procedures.

Figure 1 illustrates a simplified flow chart for the ANSYS SMART procedures for fatigue crack propagation.

## 3. Results of Numerical Simulations

### Modified Compact Tension with Different Pre-Crack Location

**Figure 2:**
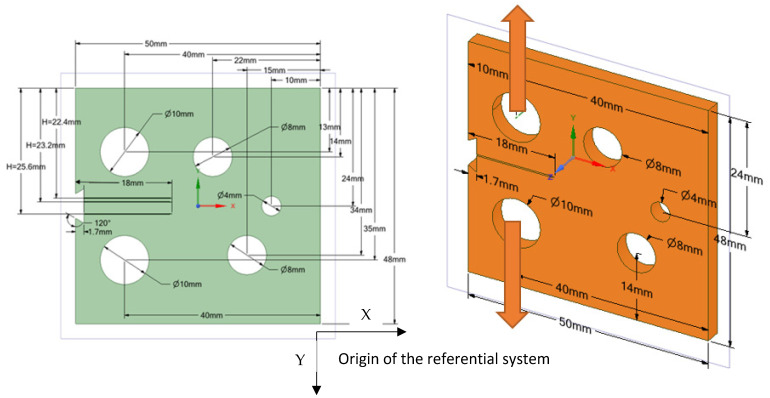
Modified compact tension geometrical dimensions.

**Figure 3:**
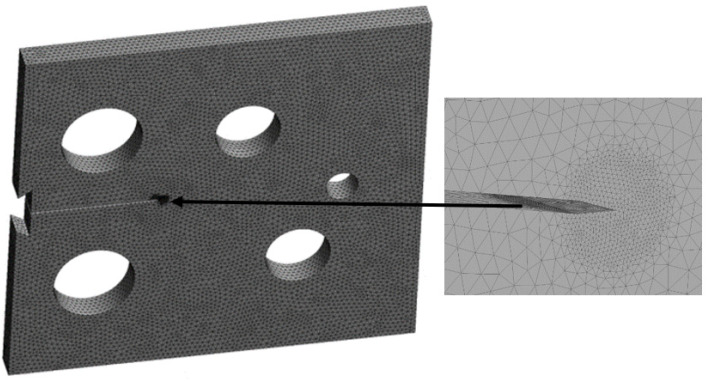
Initial mesh for the modified compact tension.

The modified compact tension specimen was studied in three distinct configurations in this study. The modified specimens vary from standard specimens in that they have three extra holes, as shown in Figure 2, which violate the standard specimens’ symmetry and result in curvilinear fatigue crack pathways. The actual crack initiation locations are compared to the nominal position of the notch tip in the geometries, as shown in Table 1. The considered material was a nickel-based superalloy with the following material properties shown in Table 1. The amount of the applied load was *p* = 3.6 kN with a stress ratio of R = 0 and cyclic frequency of 20 Hz. Changing the vertical location of the original notch (H) up or down its normal midline position, as illustrated in Table 2, leads to altering the path and ultimate destination of the crack growth. As shown in Figure 2, the vertical notch location (H) is defined relative to the geometry’s top edge. The initial mesh generated by ANSYS, which had a 1 mm element size and generated 292,160 nodes and 192,860 elements, is shown in Figure 3, which employed the sphere of influence at the crack tip area. There are three different scenarios for the crack growth trajectory based on the nominal notch positions.

**Specimen 1**

**Figure 4:**
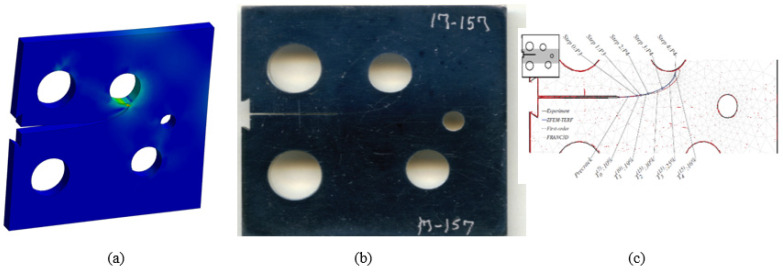
Specimen 1, crack propagation path (**a**) ANSYS results, (**b**) experimental results [46], and (**c**) numerical results [47].

The initial crack in this specimen was located at 22.4 mm from the specimen’s top edge. Comparisons of the simulated crack propagation trajectory using ANSYS to the reference experimental [46] and numerical [47] paths are shown in Figure 4a–c, respectively. Crack propagation trajectories in the numerical findings provided by [47] were predicted in three steps: the first step is to use the hyper-complex FEM trial energy response function (ZFEM-TERF) technique for crack trajectory estimation; at each step of crack growth, the model is updated with curvilinear crack path segments that are generated by the trial energy response function (TERF) approach. A finite element model was generated using the FRANC3D program in the second step before being solved using the Abaqus software in the final step. In comparison to the numerical crack growth paths presented in Figure 4c applying the ZFEM-TERF approach and FRANC3D [31], Figure 4a–b indicate that the estimated crack propagation trajectory in this study is very consistent with the experimental trajectory [46].

**Specimen 2**

**Figure 5:**
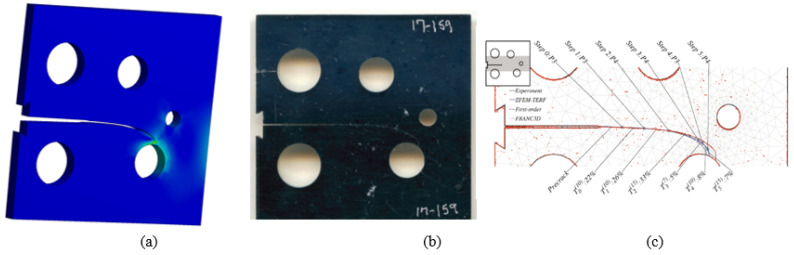
Specimen 2, crack propagation path (**a**) ANSYS results, (**b**) experimental results [46], and (**c**) numerical results [47].

The initial crack in the second specimen was located at 25.6 mm from the specimen’s top edge. The predicted crack propagation trajectory using ANSYS has matched the experimental trajectory reported by [46] more closely than the predicted trajectories estimated by [47], which had tighter curvature trajectories, as illustrated in Figure 5.

**Specimen 3**

**Figure 6:**
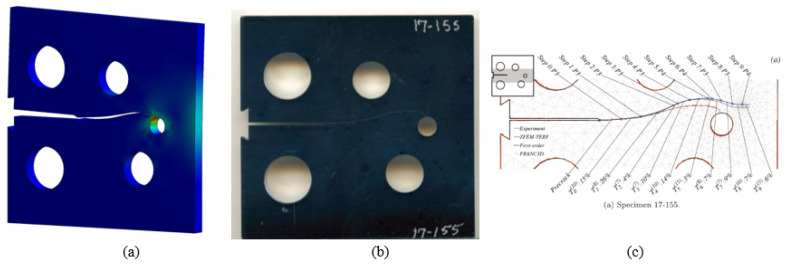
Specimen 3, crack propagation path (**a**) ANSYS results, (**b**) experimental results [46], and (**c**) numerical results [47].

The initial crack in the second specimen was located at 23.2 mm from the specimen’s top edge. As can be seen in Figure 6, the estimated crack propagation trajectory tightly matches the experimental crack growth trajectory reported by [46] compared to the predicted paths from the numerical results using ZFEM-TERF and FRANC3D conducted by [47], which deviated from the experimental path [46].

**Figure 7:**
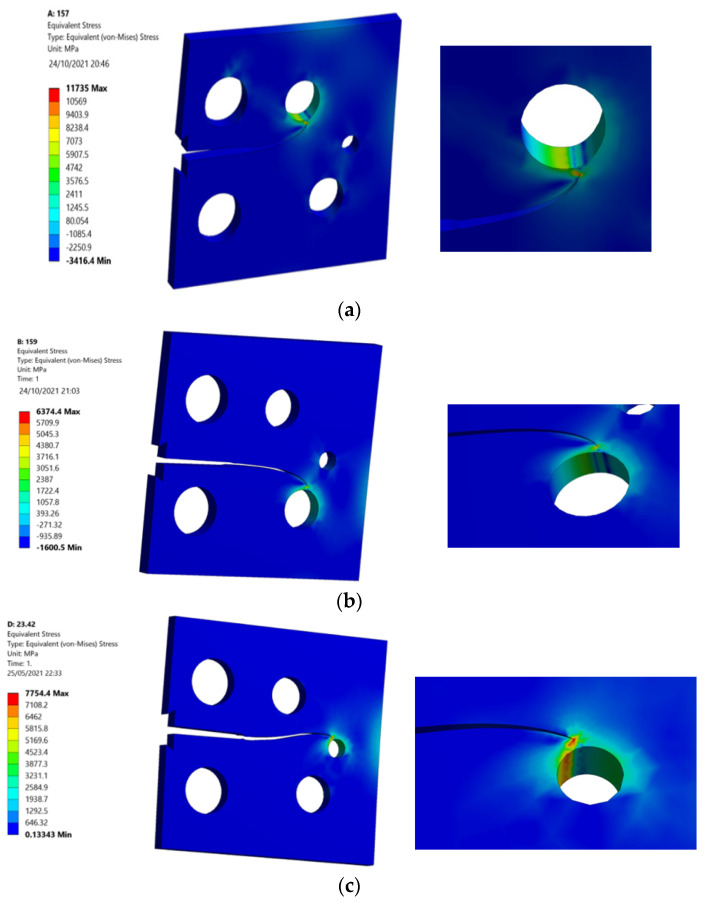
Von Mises stress distribution of (**a**) specimen 1, (**b**) specimen 2, and (**c**) specimen 3.

**Figure 8:**
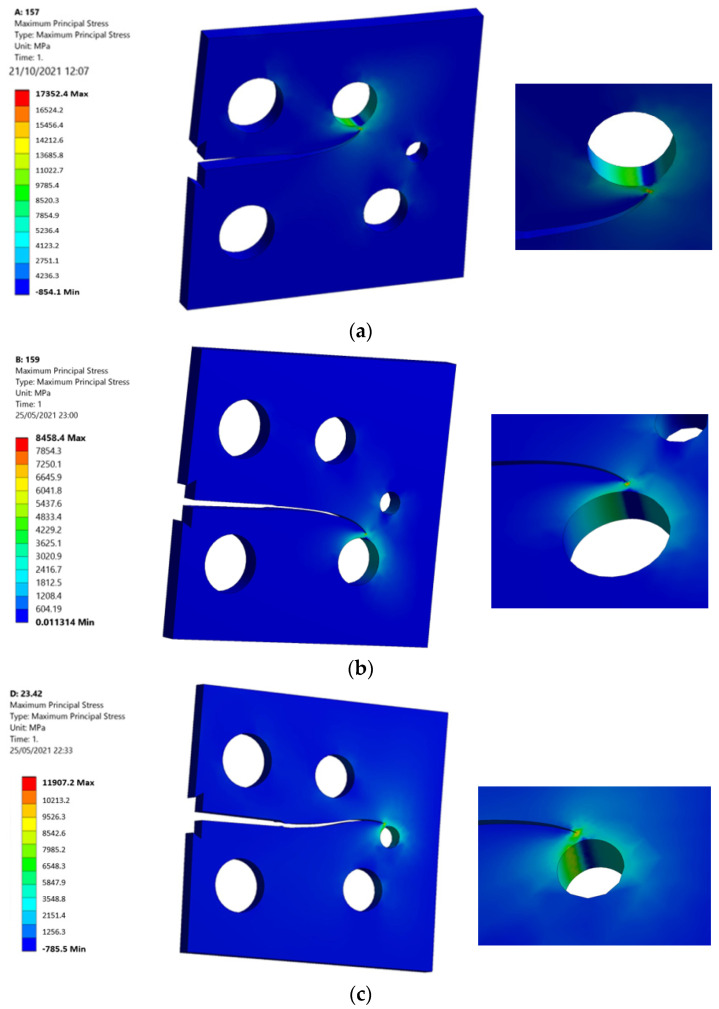
Maximum principal stress of (**a**) specimen 1, (**b**) specimen 2, and (**c**) specimen 3.

Considering that, the von Mises stress as well as the maximum principal stress are essential parameters for crack propagation assessment, which indicate the regions of maximum and minimum stresses on the geometry. Figure 7 and Figure 8 show the von Mises stress distribution stress contour as well as the maximum principal stress for each of the three specimens. The von Mises stresses and the maximum principal stress were higher in specimen one, where the top hole was located closer to the crack based on the original crack location. As the crack also sinks on the smallest hole near the right edge of the specimen, specimen two had the lowest values of the von Mises stresses and the maximum principal stress, whereas specimen three had the intermediate values of both stresses, as the crack also sinks on the second lower hole near the right edge of the specimen.

**Figure 9:**
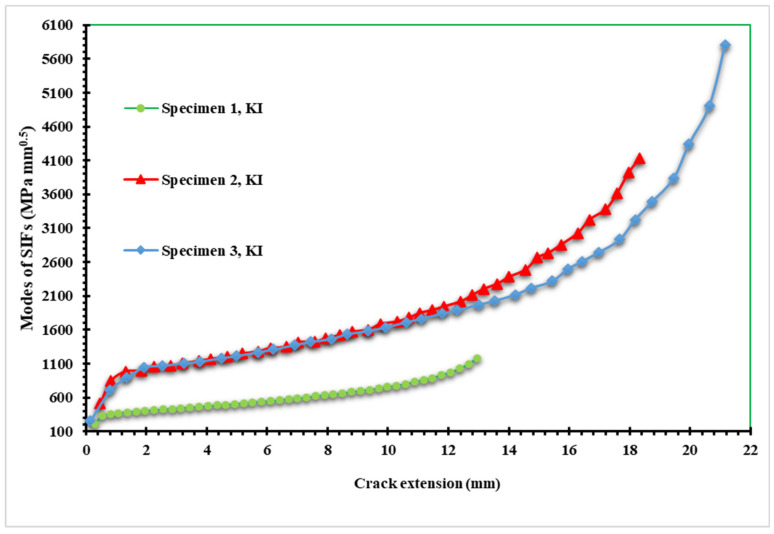
The first mode of SIFs versus crack length for the three specimens.

**Figure 10:**
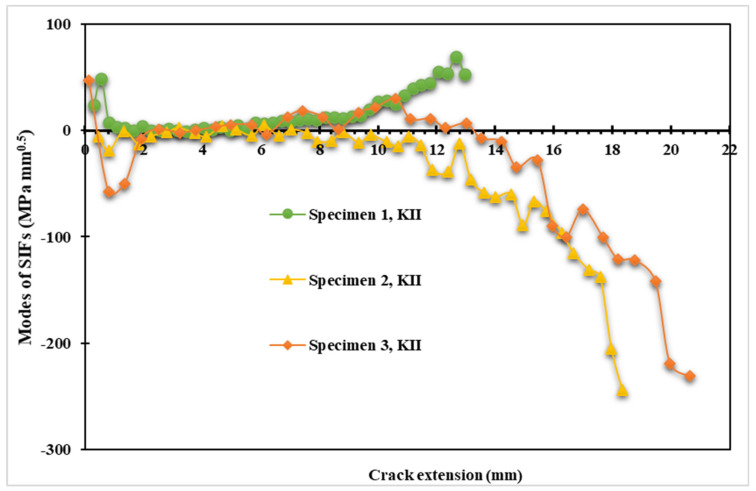
The second mode of SIFs versus crack length for the three specimens.

The results of the opening mode of SIF (*K_I_*) for the three samples are shown in Figure 9. The maximum values of *K_I_* are 1205 MPa mm^0.5^, 4136 MPa mm^0.5^, and 5800 MPa mm^0.5^ for a crack length of 12.97, 18.33 mm, and 21.161 mm for specimens one, two, and three, respectively. Similarly, Figure 10 also displays the estimated values for the second mode of stress intensity factor (*K_II_*). As the crack follows a curving trajectory toward the top hole, the *K_II_* values for the first specimen increase to a maximum of 96.133 MPa mm^0.5^ at the boundary of the hole. However, in specimens two and three, the values of *K_II_* decreased with negative values as the crack propagated on a curved path in the opposite direction of specimen one, with minimum values of −243 MPa mm^0.5^ and −230 MPa mm^0.5^ for specimens two and three, respectively. In the mixed-mode situations, the direction of the tangential component of the applied load is attributed to the negative mode II stress intensity factor. The signs of SIFs depend on the orientation of the crack with the loading.

**Figure 11:**
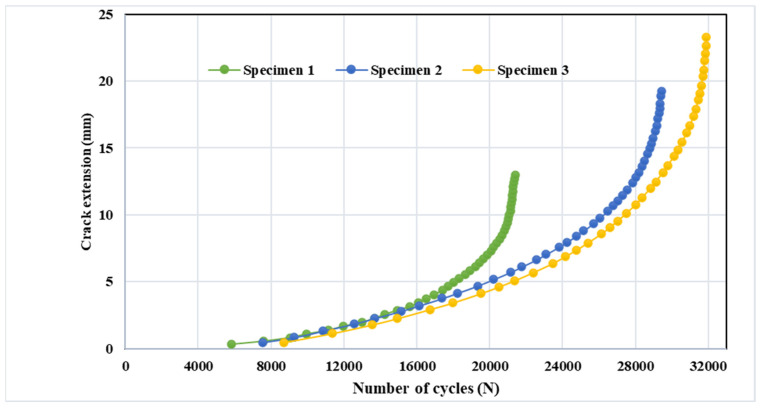
Predicted fatigue life cycles for the three specimens under stress ratio R = 0.

To determine fatigue life under constant amplitude loading circumstances with a stress ratio of R = 0, a step-by-step simulation of crack propagation was performed according to the associated SIFs. Figure 11 displays the predicted fatigue life cycles for each specimen; as seen in this figure, the fatigue life cycles were gradually increased from specimens one to three, since the stress intensity factors were also increased to the same extent for all of the specimens.

**Figure 12:**
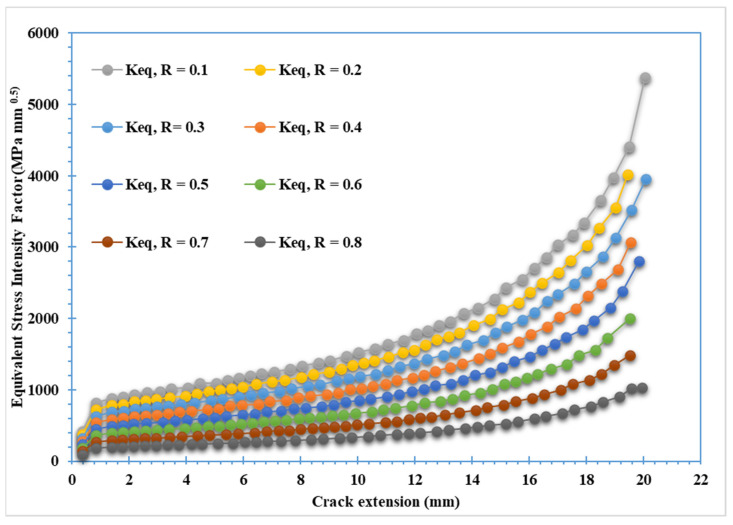
Equivalent stress intensity factor for specimen 3 with different stress ratios.

**Figure 13:**
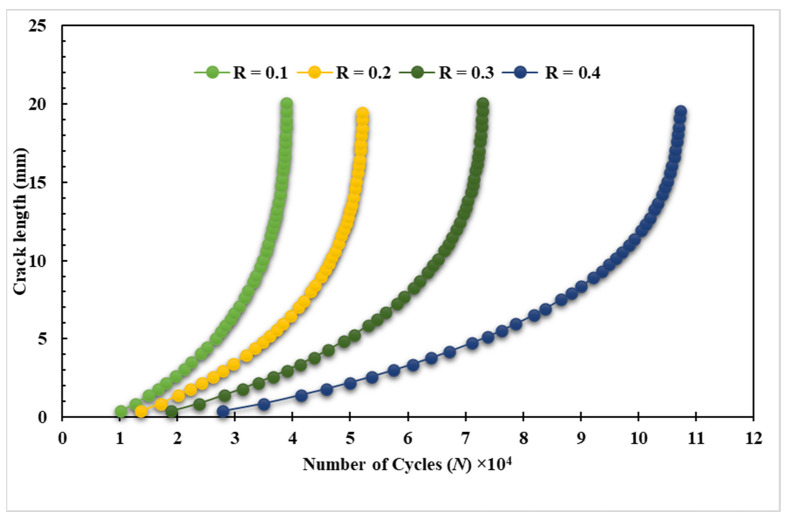
Predicted fatigue life cycles for specimen 3 with stress ratios, R = 0.1–0.4.

**Figure 14:**
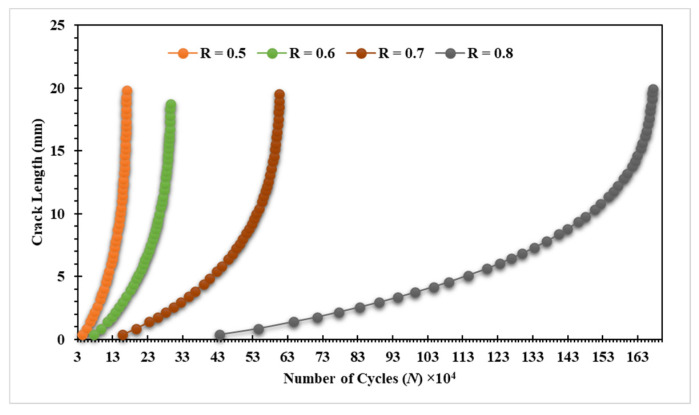
Predicted fatigue life cycles for specimen 3 with stress ratios, R = 0.5–0.8.

**Figure 15:**
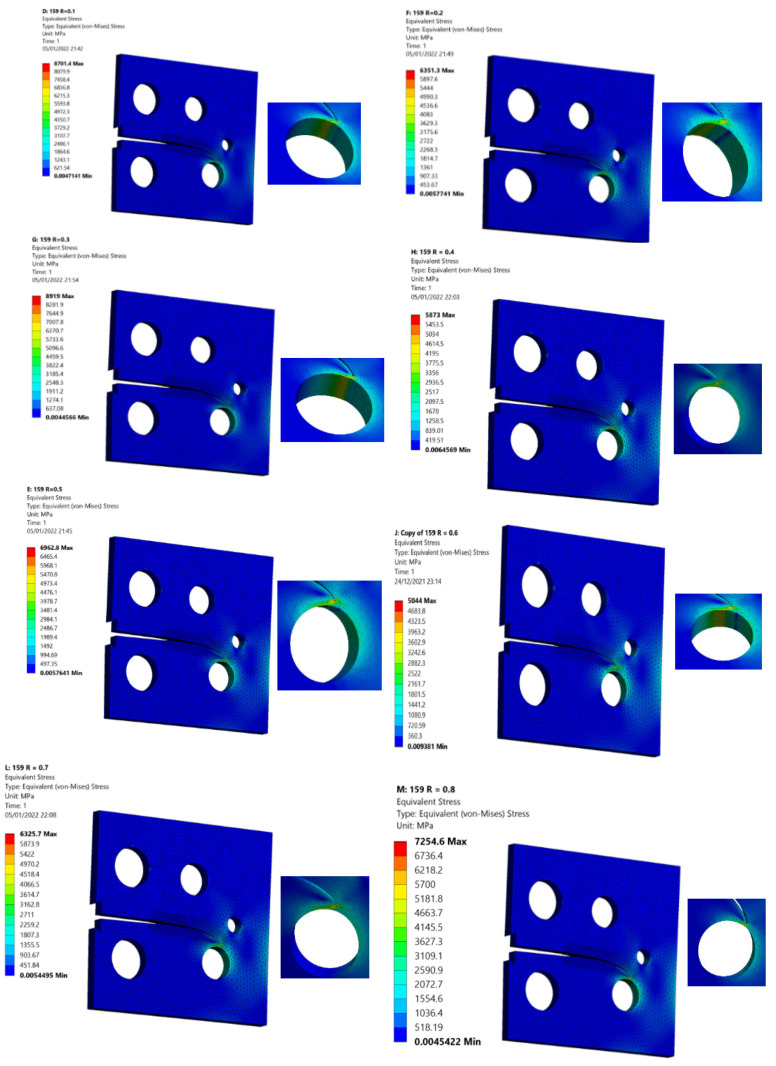
Von Mises stresses distribution for specimen 3 with various stress ratios, R = 0.1–0.8.

The third specimen was simulated at various stress ratios ranging from R = 0.1 to 0.8 to correlate the stress ratio effects on the equivalent stress intensity factor as well as fatigue crack growth rates. Almost at a given applied cyclic equivalent stress intensity, an increase in load ratio leads to an increase in fatigue crack growth rate. Equivalently, the observed equivalent stress intensity factor for fatigue crack growth decreases as the load ratio is increased, as shown in Figure 12. In other words, at high-stress ratios, less accumulated fatigue energy is necessary to support crack growth than at lower stress ratios. In contrast, the number of load cycles with respect to the crack growth extension increases as the stress ratio increases, as shown in Figure 13 and Figure 14 for the stress ratios ranging from 0.1 to 0.8. This effect is proportional to the maximum concentration of von Mises stress and hence to the driving force of mode I cracking. According to the results shown in Figure 13 and Figure 14, the percentages of increase in the equivalent stress intensity factors for different stress ratios ranging from 0.1 to 0.8 are not equal to the percentages of increase in the fatigue life cycles. Damage distributions differed depending on the stress ratios. Damage was equally distributed along with the specimens with larger stress ratios, but it was severe and concentrated at lower stress ratios (0.1–0.4), resulting in higher self-generated temperatures and specimen failure at shorter lifetimes. The von Mises stress distribution for specimen three under different stress ratios R = 0.1–0.8 is shown in Figure 15. It is found that von Mises stress decreases as the stress ratio increases, which was also related to the increase in the fatigue life cycles.

## 4. Conclusions

This study investigated the fatigue crack growth in isotropic linear elastic materials under constant amplitude loading where some defects such as holes are intentionally introduced in the material and different load ratios are applied. For this purpose, the finite element software ANSYS was used. The topic of study is certainly very important and can result in a deeper understanding of crack propagation and material design. The fatigue crack propagation of a modified compact tension specimen with various pre-crack locations was simulated using the ANSYS SMART methodology. Based on the Paris law, the crack growth simulation in SMART used tetrahedral meshes for the crack fronts that were updated automatically when the crack front was modified as a consequence of crack propagation. Based on the position of the hole and the starting position of the crack tip, the growth of the crack was either attracted to the hole and changed its trajectory to reach the hole “sink in the hole behavior” or deviated away from the hole and grew when the hole was missing “missed hole behavior”. The influence of a wide range of load ratios (R = 0, 0.1, 0.2, 0.3, 0.4, 0.5, 0.6, 0.7, 0.8) on fatigue crack growth, fatigue life, and equivalent range of SIF was investigated. According to the predicted results, it was found that as the stress ratio increased and the fatigue life cycles rapidly increased, whereas von Mises stress decreased.

## Figures and Tables

**Table 1:** Mechanical properties of the nickel-based superalloy material.

Properties	Metric Units Value
Elasticity modulus, *E*	211 GPa
Poisson’s ratio, *υ*	0.3
Yield strength, *σ_y_*	422 MPa
Ultimate strength, *σ_u_*	838 MPa
Fracture toughness, *K_IC_*	$130\;\mathrm{MPa}\;\sqrt{m}$
Paris’ law coefficient, *C*	1.02 × 10^–11^
Paris’ law exponent, *m*	2.5

**Table 2:** Pre-crack position for the modified compact tension.

Specimen Number	Crack Tip Position (mm)		
	(H)	(x)	(y)
**1**	22.4	−32	25.6
**2**	25.6	−32	22.4
**3**	23.2	−32	24.8

## Data Availability

The data presented in this study are available upon request from the corresponding author.
